# Transforming Growth Factor Beta 3 Is Required for Excisional Wound Repair In Vivo

**DOI:** 10.1371/journal.pone.0048040

**Published:** 2012-10-26

**Authors:** Mark Le, Rachelle Naridze, Jasmine Morrison, Leah C. Biggs, Lindsey Rhea, Brian C. Schutte, Vesa Kaartinen, Martine Dunnwald

**Affiliations:** 1 Department of Pediatrics, Carver College of Medicine, The University of Iowa, Iowa City, Iowa, United States of America; 2 Department of Microbiology and Molecular Genetics, Michigan State University, East Lansing, Michigan, United States of America; 3 Department of Biologics and Material Science, University of Michigan, Ann Arbor, Michigan, United States of America; University of Bergen, Norway

## Abstract

Wound healing is a complex process that relies on proper levels of cytokines and growth factors to successfully repair the tissue. Of particular interest are the members of the transforming growth factor family. There are three TGF-ß isoforms–TGF- ß 1, 2, and 3, each isoform showing a unique expression pattern, suggesting that they each play a distinct function during development and repair. Previous studies reported an exclusive role for TGF-ß 3 in orofacial development and a potent anti-scarring effect. However, the role of TGF- ß 3 in excisional wound healing and keratinocyte migration remains poorly understood. We tested the effect of TGF-ß 3 levels on excisional cutaneous wounds in the adult mouse by directly injecting recombinant TGF-ß 3 or neutralizing antibody against TGF-ß 3 (NAB) in the wounds. Our results demonstrate that TGF-ß 3 does not promote epithelialization. However, TGF-ß 3 is necessary for wound closure as wounds injected with neutralizing antibody against TGF-ß 3 showed increased epidermal volume and proliferation in conjunction with a delay in keratinocyte migration. Wild type keratinocytes treated with NAB and *Tgfb3*-deficient keratinocytes closed an in vitro scratch wound with no delay, suggesting that our in vivo observations likely result from a paracrine effect.

## Introduction

Wound healing is a complex series of overlapping events that involves inflammation, proliferation, and extracellular matrix synthesis and remodeling [1]–[3]. Amongst the multitude of cytokines and growth factors required for proper wound closure (for review [4]), transforming growth factors (TGF-ß) play an essential role. There are three TGF-ß isoforms–TGF-ß 1, 2, and 3–that share approximately 80% structural homology in their active regions. TGF-ß 1, 2, and 3 bind to the TGFßR2 leading to the recruitment and phosphorylation of TGFßR1, which in turn phosphorylates receptor-regulated SMADs. These SMADs can then bind the co-smad 4, and this complex accumulates in the nucleus to activate target genes [5], [6]. Despite their common signaling pathway and structural homologies, each isoform shows a unique expression pattern [7]–[9], suggesting that they each have a distinct function during development [10]. TGF-ß1 knockout mice die shortly after birth because of a wasting syndrome and multifocal inflammatory reaction, while TGF-ß2 knockout mice display a mixture of cardiovascular and musculoskeletal abnormalities [11], [12]. Lastly, mice with a mutation in the TGF-ß3 gene exhibit abnormal lung development and cleft palate, and are neonatal lethal [13], [14].

As embryonic development and wound healing share many molecular pathways, it is not surprising that a TGF-ß isotype-specific distinction is also observed during tissue repair. For example, TGF-ß1 is highly expressed in both compartments only after the initiation of epithelialization, while TGF-ß3 appears upregulated early in the process, particularly in the migrating epidermis [15]. Additionally, excessive production of TGF-ß1 and – ß2 isoforms promotes scar formation while the addition of TGF-ß3 reduces scarring [16], [17]. Unfortunately, it has been difficult to assess the specific function of the TGF-ß isoforms on wound healing in vivo because of lack of survival of the knockout animals. Only the effect of TGF-ß1 on wound healing has been directly tested using the TGF-ß1 knockout mouse, animal that exhibit major delays in each of the phases of the healing process [18]. Using multiple in vivo murine models with global loss of TGF-ß signaling, several studies showed altered wound healing with accelerated epithelialization and impaired inflammatory response [19]–[21].

We were particularly interested in TGF-ß3. Mice deficient for TGF-ß3 exhibit cleft palate and abnormal lung development [13], [14] yet do not have any other apparent phenotype. However, using grafts of embryonic *Tgfb3*-deficient skin, Li et al. showed that only TGF-ß3, and not the other TGF-ß isoforms, protected keratinocytes from TPA-induced cell death [22], suggesting that TGF-ß3 may not be required for epidermal homeostasis, but necessary under repair conditions. In vitro studies exposing keratinocytes, fibroblasts and endothelial cells to TGF-ß3 indicated a selective inhibition of dermal, but not epidermal, cellular migration [23]. Its best-known function is the prevention of scar formation in animals and humans [17], [24]–[26], a role that was established using incisional wound healing models that require minimal epithelialization. However, the role of TGF-ß3 in excisional wound healing and keratinocyte migration remains poorly understood. In our current study, we tested the effect of TGF-ß3 levels on excisional cutaneous wounds in the adult mouse by directly injecting recombinant TGF-ß3 or neutralizing antibody against TGF-ß3 in the wounds. Our results demonstrate that TGF-ß3 does not promote epithelialization, but is necessary for wound closure in vivo.

## Materials and Methods

### Animals

All animal procedures were approved by the University of Iowa Institutional Animal Care and Use Committee protocol 1105108. Male and female (10–16 wk) C57Bl/6, *Tgfb3*-Cre [27], and Rosa 26-LacZ (*R26R-LacZ*; Jackson Laboratories, Bar Harbor, ME, USA) were used. Embryonic days (E)17.5 embryos were used to extract keratinocytes for cell culture studies as previously described [28].

### Cutaneous Wounds

Two six mm diameter full-thickness punch cutaneous wounds were made on the dorsorostral skin of C57Bl/6 or *Tgfb3-Cre*;*R26R-LacZ*. One day later, 25 µl of saline, IgG control (2.5 µg, R&D systems, Minneapolis, MN), TGF-ß3 (50 ng, R&D systems), neutralizing antibody against TGF-ß3 (NAB; 2.5 µg, R&D systems), or a combination of both (NAB and TGF-ß3 were incubated together for 1 h prior in vivo injection to insure the neutralization of exogenous TGF-ß3) was injected under each wound. Four, seven and eleven days post-wounding, animals were sacrificed and wounds harvested. Left wounds were fixed in 4% paraformaldehyde and embedded in paraffin for histological and immunological analysis. Right wounds were embedded in OCT for cryopreservation and immunological analysis. For any given experiment, only one wound per animal was used.

### Morphometric Analysis

**Figure 1:**
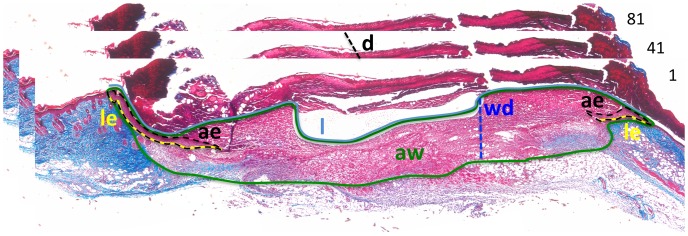
Schematic of the morphometric analysis. l = length of the wound (blue solid line); le = length of the epidermis (yellow solid line); ae = area of the epidermis (black dotted line); aw = area of the granulation tissue (green solid line); wd = wound depth (blue dotted line); d = distance between two sections (black dotted line). Note that in case of closed wound, le = l; in case of non-epithelialized wounds (this example), le<l. In this example, every 40 sections were analyzed (see numbers on the top right corner of each picture), so d = 280 µm.

Serial sectioning (7 µm thick) of each wound was performed. Several morphometric parameters were measured every 10 to 40 sections (d = distance between two sections) using NIH-ImageJ as shown in Figure 1: the length of the wound (l), the length of the epidermis (le), the area of the epidermis (ae) and the area of the granulation tissue (aw). From these measurements, other metrics were calculated: the overall epidermal area covering the wound (ea = sum of (le×d)), the overall wound area (wa = sum of (l×d)), the epidermal volume (ev = sum of (ae×d)) and the wound volume (wv = sum of (aw×d)). The percentage of closure was calculated as the ratio of epidermal area over wound area. The length of the epidermal migrating tongue (lmt) and the depth of the wounds were measured on three sections in the middle of the wound only and presented as average of the three measurements for each wound.

### Immunostaining

Paraffin embedded sections were immunostained as previously described [28]. Primary antibodies were: mouse monoclonal against Proliferative Cell Nuclear Antigen (PCNA, Biomeda, Foster City, CA), alpha-smooth muscle actin (Sigma, St-Louis, MO), and beta-actin (Sigma); rabbit polyclonal against Interferon Regulator Factor 6 (Irf6 [29]). Secondary antibodies were: goat anti-mouse FITC (Sigma), and goat anti-rabbit Alexa 568 (Molecular Probes, Grand Island, NY). Nuclear DNA was labeled with DAPI. For PCNA staining, antigen retrieval in citrus buffer was performed. Microscopic observations were performed with an E800 Eclipse Nikon and photomicrographs recorded in black and white with an RT-Slider (SPOT), merged and pseudocolorized. Keratinocytes were immunostained with rat monoclonal anti-BrdU antibody (Abcam, Cambridge, MA) according to the manufacturer’s instruction, followed by immunodetection with a donkey anti-rat Alexa 488 (Molecular Probes).

### Cell Culture and Scratch Wound Assay

Keratinocytes from E17.5 embryonic skin were isolated and cultured as previously described [28], [30]. For proliferation assay, keratinocytes were grown on collagen IV coated coverslips for 48 h and 5-bromo-2′-deoxyuridine-5′-triphosphate (BrdU, 10 µM) was added two hours before cells were fixed in methanol-acetone (70%/30% v/v) for immunostaining according to the manufacturer’s instruction and previously described [28].

At confluency, a scratch in the middle of the dish was performed with a yellow tip. Three independent fields were photomicrographed before, immediately after, and 12 and 24 h post-scratch. The open area of the scratch was measured with NIH-ImageJ and the percentage of closure calculated.

### Xgal Staining

X-gal staining was performed as described [28].

### Data Analysis and Statistics

Between group comparisons were performed by one way ANOVA followed by Bonferroni’s honestly significant difference post-hoc tests. Student t-test was performed when only two groups were compared. P<0.05 was considered statistically significant. All data are presented as average with error bars indicating the standard error of the mean.

## Results

### Tgfb3 is Expressed in the Upper Layers of the Epidermis Throughout the Wound Healing Process

**Figure 2:**
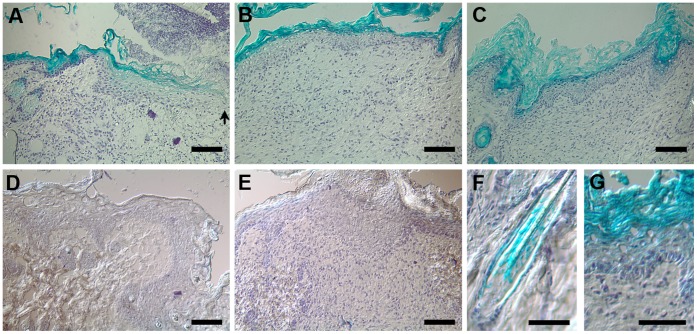
Tgfß3-Cre induced recombination in the suprabasal layers of the epidermis during wound healing. Six-mm excisional punch wounds sections were performed in *Tgfb3-Cre*;*R26R-LacZ* (a–d, f, g) or wildtype animals (e) and harvested 4 (a, d), 7 (b, e) and 11 (c) days postwounding. Tissue sections were stained for X-gal (a–c, e–g) or incubated in PBS control (d). Black arrow in (a) indicates the leading edge of the migrating keratinocytes. Note the presence of X-gal staining in the inner root sheath of the hair follicle (f) and in the subrabasal layer of the epidermis (g). Scale bar (a–e) = 100 µm; scale bar (f, g) = 50 µm.

Despite the report of the expression of TGF-ß3 throughout the epidermis [15], [22], we failed to detect the presence of Tgfb3 using commercially available antibody on tissue from murine wounds. Alternatively, we took advantage of a new *Tgfb3*-Cre knock-in allele [27], [31] and crossed it with the R26R-LacZ reporter mouse [32]. X-gal staining showed strong LacZ expression in the upper layers of the epidermis (Figure 2a–c, close-up in g), as well as in hair follicle (Figure 2f) and sebaceous gland as previously reported [31]. This pattern of expression is similar to the expression of Cre-recombinase as previously described [31]. During wound healing, X-gal was detected in suprabasal layers of the migrating keratinocytes 4 days post-wounding (Figure 2a), while it was restricted to the superficial layers of the newly formed epidermis 7 and 11 days post-wounding (Figure 2b, c). This staining was specific as transgene negative animals showed no X-gal positive signal (Figure 2e).

### The Absence of TGF-ß3 Impairs Wound Closure

**Figure 3:**
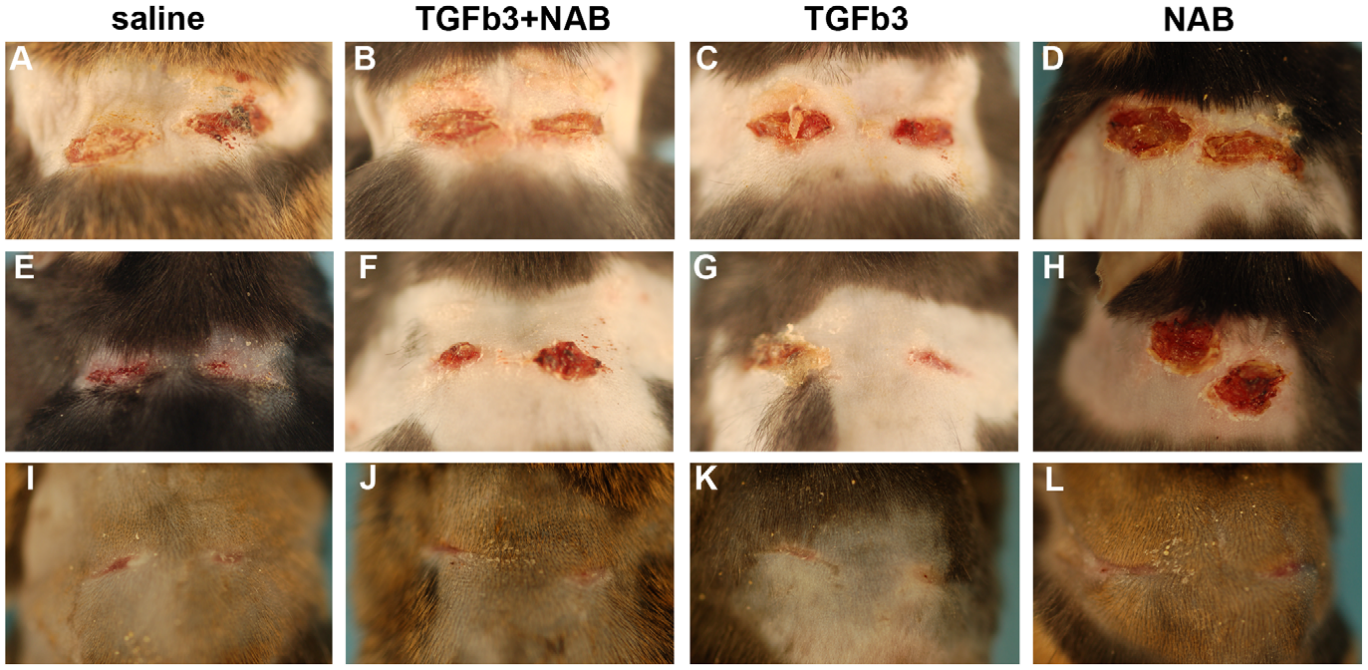
Macroscopic photomicrographs of excisional wounds. Six-mm excisional punch wounds were performed on the back of wild type mice. One day later, wounds were treated with saline (a, e, i), TGF-ß3 and neutralizing antibody (NAB) against TGF-ß3 (b, f, j), TGF-ß3 (c, g, k), and NAB (d, h, l). Wounds were harvested 4 days (a–d), 7 days (e–h) and 11 days (i–l) post-wounding.

In order to assess the requirement for TGF-ß3 during wound healing, we performed excisional wounds on the back of wild type mice and followed their healing over an 11-day period (Figure 3). We used macroscopic photomicrographs as a first indicator of wound healing. Four days post-wounding, healing was engaged with the presence of scab and redness in the wound. TGF-ß3 -treated and control wounds (saline injected and TGF-ß3+NAB injected) appeared about the same size, yet the wounds treated with TGF-ß3 neutralizing antibody (NAB) were slightly larger (Figure 3 a–d). At seven days, control wounds (Figure 3 e, f) appeared identical to wounds injected with TGF-ß3 (Figure 3 g). However, wounds treated with NAB alone were redder and larger than the other three groups (Figure 3 h). No difference was noticeable 11 days post-wounding, time when all the wounds were closed (Figure 3 i–l).

**Figure 4:**
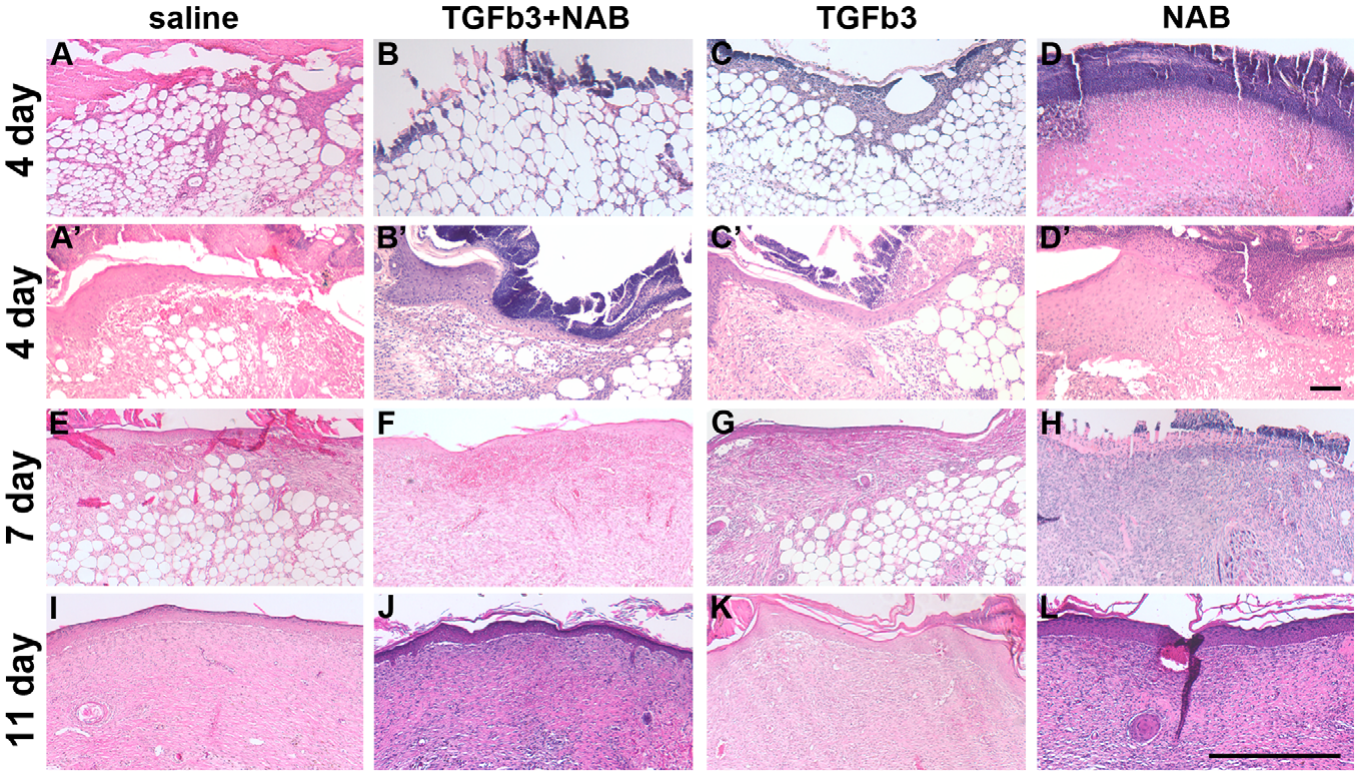
Histological features of excisional wounds. Hematoxylin and eosin staining of the section in the middle of the wound is shown as representative of each treatment group (saline, a, e, i; TGF-ß3+NAB, b, f, j; TGF-ß3, c, g, k; NAB, d, h, l) and time points (4 days post-wounding, a–d; 7 days post-wounding, e–h; 11 days post-wounding, i–l). Only the middle of the wound of each section is shown. Scale bar = 500 µm.

To confirm our macroscopic phenotype, we performed histological analysis of these same wounds. Figure 4 shows the histological features of the middle of the wound of each group at the different time points. All wounds were open four days post-wounding (Figure 4 a–d). At seven days, wounds were closed in controls (IgG control not shown) and TGF-ß3 -injected wounds, while epithelialization was incomplete in NAB-injected wounds (Figure 4 e–h). All of the wounds were covered by an epithelium 11 days post-wounding (Figure 4 i–l).

**Figure 5:**
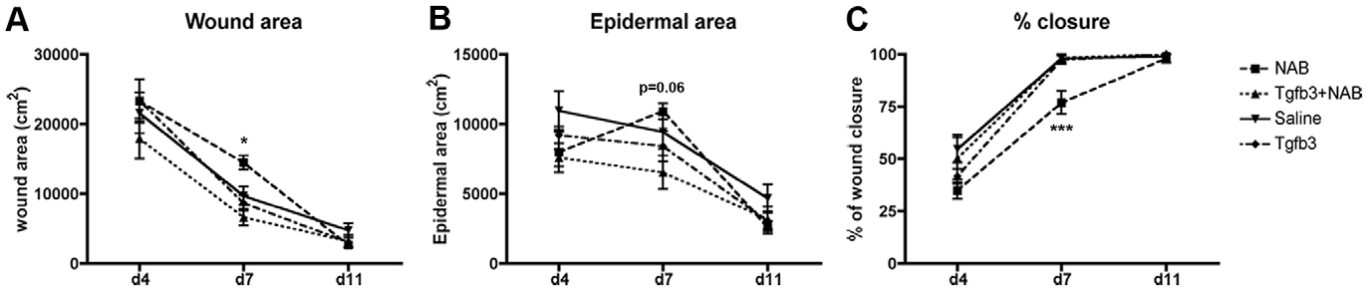
TGF-ß3 is required for proper wound closure in vivo. Morphometric analysis of serial histological sections and calculation of the total wound area (a), the epidermal area (b) and the percentage of wound closure (c). N = 6−8 per group. *P<0.05, ***P<0.001.

Quantification of the percentage of closure was performed using morphometric analysis of the entire wound, and not data from the middle of the wound only (Figure 5). As described in detail in the method section, wound area and epidermal area were calculated for each wound (Figure 5 a, b). The percentage of closure was identified as the ratio of the total epidermal area over the wound area (Figure 5 c). Already at four days post-wounding, the NAB-treated wounds had the lowest percentage of closure, yet the data was not significant. Morphometric measurements of seven-day wounds confirmed the macroscopic observations and indicated that NAB-treated wounds were 75% closed while all the other treated groups were completely epithelialized. All groups were closed eleven days post-wounding.

In order to insure that our findings were not due to inhibited wound contraction, we measured the distance between the two edges of the panniculus carnosus (Table 1). Our results show a decrease in this distance between day 4 and day 7 post-wounding (P<0.0001), but we did not find a difference amongst the treatment groups. Therefore, our results suggest that the delayed wound closure observed is due to a reduced keratinocyte migration rather than an effect of inhibited wound contraction. Together, these data indicate that TGF-ß3 is necessary for proper epithelialization, yet exogenous TGF-ß3 did not increase the rate of closure.

**Table 1:** Distance between the edges of the panniculus carnosum.

Treatment group	Day 4 post-wounding	Day 7 post-wounding^a^
Saline	5.07±0.39^b^	3.31±0.19
TGF-ß3+NAB	5.80±0.39	3.39±0.27
TGF-ß3	5.25±0.23	3.49±0.18
NAB	5.44±0.30	3.52±0.31

a Data from day 7 are significantly different than data from day 4 after t-test (***P<0.0001).

b Data amongst treatment groups is not significantly different at either days post-wounding.

### The Absence of TGF-ß3 Delays Keratinocyte Migration and Increases Cellular Proliferation in vivo

**Figure 6:**
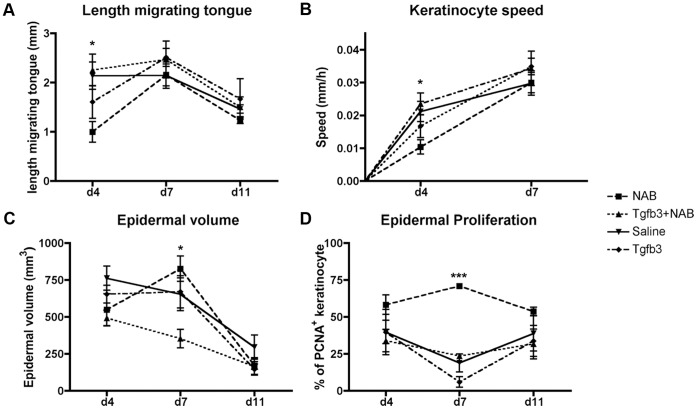
TGF-ß3 is required for proper keratinocyte migration and proliferation in vivo. (a) Length of the migrating tongue in the middle of the wound. (b) Speed of migration of keratinocyte in vivo was calculated (distance of the migrating tongue divided by the time). Morphometric analysis of serial histological sections was used to calculate the epidermal volume (c). Percentage of PCNA positive basal keratinocytes (d). N = 4−8 per group. *P<0.05, ***P<0.001.

To investigate further the wound closure defect observed in NAB-treated wounds, we asked whether the failure of epithelialization was due to a defect in migration and/or in proliferation of keratinocytes. We measured the length of the epithelial migrating tongue on three sections flanking the middle of the wound (Figure 6). Our results show the shortest migrating tongue in NAB-treated wound throughout the course of the wound, yet the data was significant only four days post-wounding (Figure 6 a). During these first 4 days, the migratory speed of keratinocytes was significantly reduced in the absence of TGF-β3 compared to the three other groups (Figure 6 b), suggesting that the reduction in the migration rate is responsible for the reduced epithelialization. Despite a shorter distance covered by the epithelial cells, the overall epidermal volume was significantly larger seven days post-wounding in NAB-treated wounds compared to the other groups (Figure 6 c). We performed immunostaining for Proliferative Cell Nuclear Antigen (PCNA, Figure 6 d), a marker of cellular proliferation, and calculated the percentage of positive keratinocytes in the basal layer of the migrating tongue. Our results show an overall increase in keratinocyte proliferation in NAB-treated wounds that was significant at 7d (Figure 6 d) compared to control. Together, these in vivo data show that in the absence of Tgfb3, keratinocytes exhibit an increased proliferation rate as well as a reduced migratory capacity, which leads to a delay in epithelialization.

Although the addition of exogenous TGF-ß3 did not have an apparent effect on wound closure, we observed a significant decrease in epidermal volume. Accordingly, the percentage of PCNA positive keratinocytes was the lowest amongst the groups (Figure 6 d). These data indicate that the addition of TGF-ß3 reduced the rate of proliferation of keratinocyte without affecting the epithelialization.

### Tgfb3 is Not Required for Scratch Wound Closure in vitro

**Figure 7:**
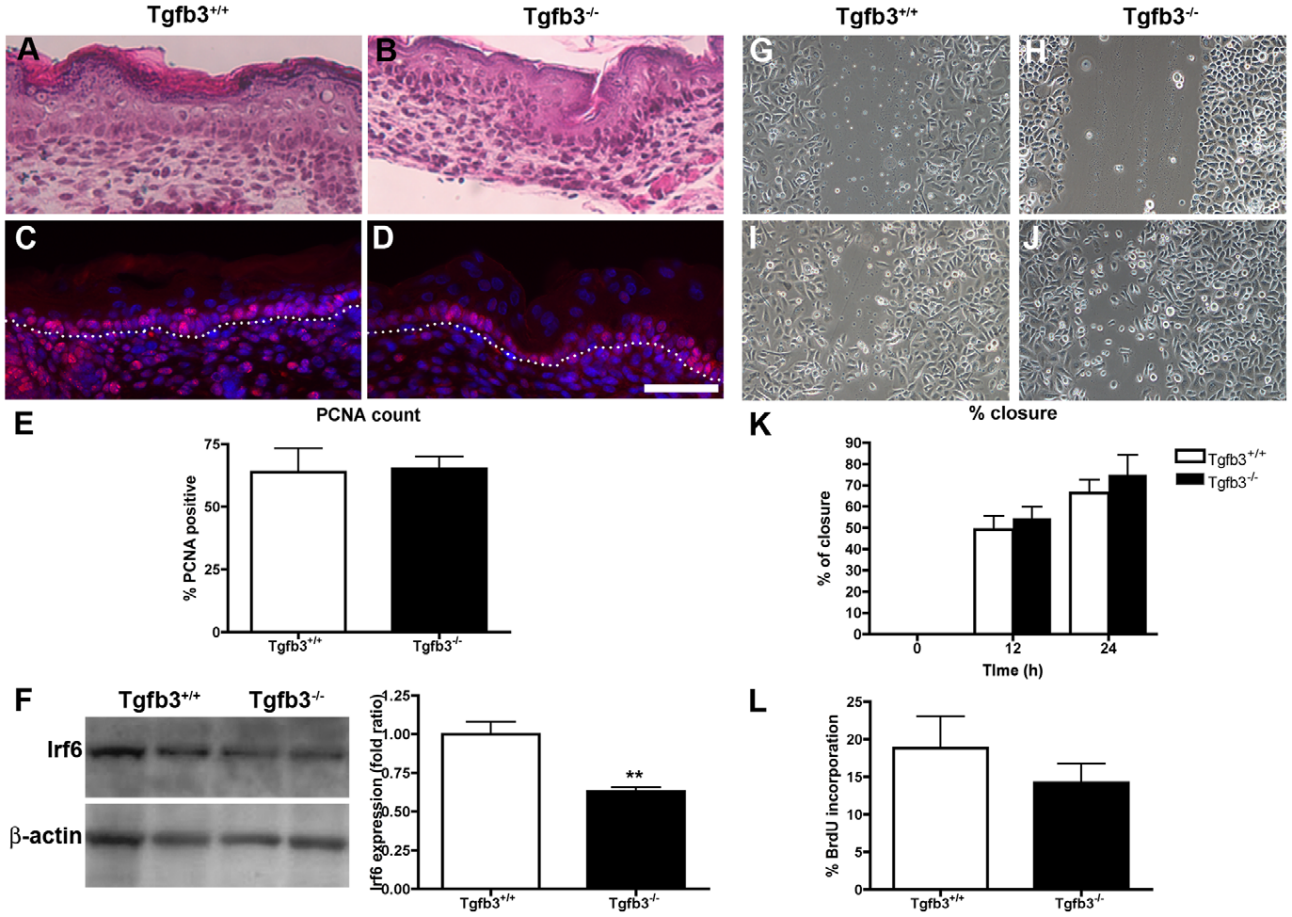
TGF-ß3 is not required for epidermal homeostasis and in vitro scratch wounds. Histological sections of skin from E17.5 wild type (a) and *Tgfb3*-deficient (b) embryos stained with hematoxylin and eosin. Similar sections (c, wild type; d, *Tgfb3*-deficient) were immunolabeled for PCNA (red) while nuclei were labeled with DAPI (blue). Dotted line indicates the basement membrane. Percentage of basal cells that were PCNA positive was calculated for both genotypes (e). (f) Interferon Regulatory Factor 6 (Irf6) expression in E17.5 cutaneous extracts of wild type and *Tgfb3*-deficient animals (2 samples are represented out of 6 tested). B-actin was used as loading control and densitometry used to quantify the level of Irf6 expression. (g–j) Keratinocytes were extracted from E17.5 skin and plated in culture. At confluence, in vitro scratch wounds were performed and followed over time (g, h: time point 0 h; I, j: time point 12 h). Percentage of closure was calculated for each time point (k). N = 3 per group. (f) Percentage of cells incorporating BrdU after a 2 h pulse was calculated. Scale bar = 50 µm.

In order to test whether the increase in migration and proliferation observed in the absence of TGF-ß3 during in vivo wound healing was due to tissue injury or was already present in animals lacking TGF-ß3, we took advantage of a new *Tgfb3* allele [33] and evaluated the proliferation and migration of *Tgfb3*-deficient keratinocytes (Figure 7). The keratinocytes were obtained from wild type animals or animals homozygotes for an allele where a cassette with a promoterless Cre gene replaced the coding region of the ATG containing exon 1 of the *Tgfb3* gene [33]. As a consequence, only the *Tgfb3* gene was knockout, leaving intact the other Tgfb isoforms. Previous studies with Tgfb3 knockout mice [13], [14] reported normal cutaneous homeostasis in skin grafted on the back of nude mice, yet cell death was increased upon treatment with a tumor promoting agent [22]. We did not detect morphological differences in E17.5 skin from wild type and *Tgfb3*-deficient animals (Figure 7 a, b), including proliferation in the basal layer in vivo (Figure 7 c–e) and BrdU incorporation in vitro in *Tgfb3*-deficient keratinocytes or wild type keratinocytes treated with NAB (Figure 7 l and data not shown). However, the level of Interferon regulatory factor 6 (Irf6), a transcriptional regulator of epidermal proliferation and differentiation downstream of *Tgfβ3* in oral keratinocytes [28], [34]–[36], was decreased in E17.5 skin from *Tgfb3*-deficient animals compared to wild type (Figure 7f). Scratch wounds performed in confluent wild type and *Tgfb3*-deficient keratinocyte cultures as well as wild type keratinocytes treated with NAB closed at the same rate, suggesting that TGF-β3 was not required for proper closure in vitro (Figure 7 g–k and data not shown). Of note, keratinocytes in vitro and keratinocytes epithelializing in vivo wounds migrated at similar speeds (10^−1^ mm/h). Together our data indicate that keratinocytes deficient for *Tgfb3* migrate properly in vitro, suggesting that the migration defect observed in vivo may be the result of a paracrine effect from the underlying dermis.

### TGF-ß3 is Required for Granulation Tissue Maturation

**Figure 8:**
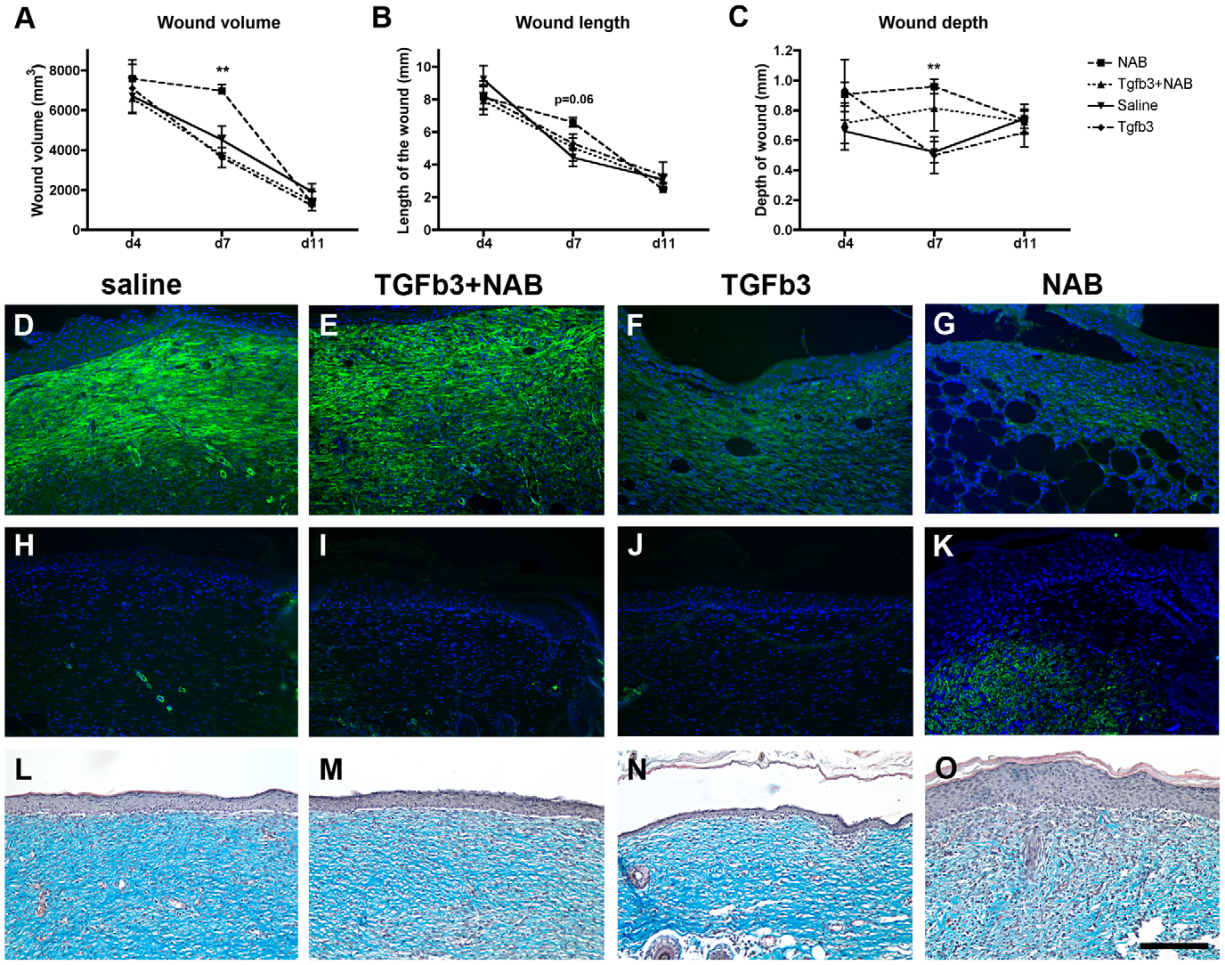
TGF-ß3 is required for granulation tissue repair. Morphometric analysis of serial sections was used to calculate the wound volume (a). Wound length (b) and depth (c) were measured on sections in the middle of the wound. N = 6−8 per group. **P<0.01 (d–k) Sections of 7-day (d–g) and 11-day (h–k) wounds were immunostained for alpha-smooth muscle actin (green). Nuclear DNA was labeled with DAPI. Sections of 11-day wounds were stained with Masson’s trichrome (l–o). Panels d–o show the middle of the wound. Scale bar = 100 µm.

TGF-ß3 has been proposed as a prophylactic anti-scarring agent for incisional wounds [25]. We evaluated the effect of TGF-ß3 levels on the granulation tissue maturation and overall wound size in our excisional wound model. In control groups, the wound volume decreases as healing progresses over time (Figure 8). The addition of exogenous TGF-ß3 did not affect the wound volume compared to controls (Figure 8 a). However, the normal decrease in wound volume that occurs as healing progresses was delayed in the absence of TGF-ß3 (Figure 8 b). A change in wound volume could be due to alteration in the width of the wound or the depth of the wound. We measured both and found a significant decrease in wound depth (Figure 8c), but only minor alterations in the length of the wound (p = 0.06).

The repair of dermal tissue results from an intricate balance between the production of extracellular materials and the activation of fibroblasts into myofibroblasts. We evaluated the expression of α-smooth muscle actin, a marker of myofibroblasts [37], [38] and found expression in arterioles in non-wounded healthy tissue flanking the wound (data not shown). Four days post-wounding, no α-smooth muscle actin was detected in wounds regardless of the treatment group (data not shown). However, seven days post-wounding, we observed robust expression of α-smooth muscle actin in the granulation tissue of control wounds (Figure 8 d, e) that decreased to an undetectable level eleven days post-wounding (Figure 8 h, i). This pattern of expression for α-smooth muscle actin was also observed in wounds treated with exogenous TGF-ß3. However, we observed a different pattern in wounds treated with NAB. Levels of α-smooth muscle actin did not decline at eleven days post-wounding. Rather, levels remained steady in NAB-injected tissues (Figure 8 k).

To investigate further the healing and scarring process, we perform Masson’s trichrome staining on samples taken eleven days post-wounding (Figure 8 l–o). Our data show the presence of collagen fibers in the granulation tissue (indicated by the blue color) of controls and TGF-ß3 treated animals. However, animals treated with NAB presented a lack of collagen fibers immediately below the epidermis, and an overall less organized network of fibers compared to the other groups (Figure 8 o). Together, these results suggest that α-smooth muscle actin expression and collagen fiber network are intimately correlated with a proper level of TGF-ß3, ultimately affecting wound volume and depth.

## Discussion

In this study, we demonstrate the requirement for proper levels of TGF-ß3 in excisional wound healing in the mouse. Exogenous addition or specific inhibition of TGF-ß3 affects different phases of healing, with profound effect on keratinocyte proliferation and myofibroblasts. Our data suggest that the migration delay and increased proliferation of keratinocytes in the absence of TGF-ß3 in vivo is non-cell autonomous, and potentially mediated by a paracrine effect.

To evaluate whether the levels of TGF-ß3 affect excisional wound healing in a murine model, we injected a well-established neutralizing antibody against TGF-ß3, as well as recombinant TGF-ß3 under the wounds. Initial macroscopic evaluation of wounds indicated a delay in healing in the presence of neutralizing TGF-ß3 antibody seven days post-wounding, while the addition of TGF-ß3 appeared to have no effect. Detailed morphometric analyses allowed us to thoroughly evaluate the effect of TGF-ß3 on the different cutaneous compartments and phases of wound healing. The most striking finding of this examination was that in the absence of TGF-ß3, wounds were not epithelialized seven days post-wounding, despite an augmented epidermal volume. Proliferation and migration are the two main biological processes governing keratinocyte behavior during wound healing. We evaluated both in our system and both were altered in the absence of TGF-ß3 in vivo.

Keratinocyte migration was delayed in the initial phase of epithelialization in vivo in the absence of TGF-ß3. However, our in vitro scratch wound assay confirmed the ability of *Tgfb3*-deficient keratinocytes to migrate at a similar rate than wild type cells, consistent with a previously described report [24]. TGF-ß3 signals through TGFβR2 and has been shown to act as a “traffic control” molecule in wounds because of its absence in plasma, and its increased level in serum [23]. It is the level of TGFβR2 that makes cells responsive to TGF-ß3 and governs their migratory behavior. Keratinocytes express low levels of TGFßR2 compared to mesenchymal cells, in tissue culture as well as in vivo during tissue repair (data not shown), rendering them fairly insensitive to the level of TGF-ß3. Although we did not evaluate the expression of TGFßR2 in *Tgfb3*-deficient keratinocytes, we would expect it to be similar between the two populations. Levels of integrin and composition of the extracellular matrix are other key modulators of keratinocyte migration, and could potentially be influenced by the level of TGF-ß3 [39], [40].

The increase in keratinocyte proliferation in the absence of TGF-ß3, and the decreased proliferation in the presence of exogenous TGF-ß3 is consistent with an overall antiproliferative effect on keratinocytes associated with TGF-ß family members [6], [41]. Although Irf6, a new transcriptional regulator of epidermal proliferation, is decreased in skin from *Tgfb3*-deficient embryos compared to wild type, it is not significantly altered in cutaneous wounds (data not shown). It stands to reason that an increased rate of proliferation in the epidermal compartment leads to faster epithelialization as reported for models of altered TGF-ß signaling in wounds [19], [21]. However, wounds treated with neutralizing TGF-ß3 antibody were still open seven days post-wounding. This observation is reminiscent of an overall increase in epidermal proliferation in human non-healing chronic wounds [42], and particularly in cells at the leading edge. An attractive hypothesis is that proliferation is only beneficial to epithelialization if present at the initial wound margin away from the leading edge, and detrimental if detected in cells at the leading edge. Interestingly, we did not detect altered proliferation in *Tgfb3*-deficient keratinocytes and wild type keratinocytes grown in the presence of NAB, as well as in the basal layer of embryonic skin, suggesting that the proliferation defect in injured skin may be unique to a condition of tissue repair. Furthermore, it supports a role for a TGF-ß3-dependent paracrine effect on keratinocytes, mediated by cells from the granulation tissue. Recent reports have identified a TGFßR2-Smad-independent TGF-ß3 signaling in palatogenesis [43]. This non-canonical pathway utilizes the MAPK signaling, known to regulate the production of numerous downstream targets, including interleukin 6 [44], a well-known critical regulator of keratinocyte migration [45], [46].

TGF-ß3 is probably best-known for its antiscarring effect [24], and recombinant TGF-ß3 has been used in clinical trials as prophylactic treatment of human scars [25]. TGF- ß3-injected wounds exhibit decreased expression of α-smooth muscle actin in the granulation tissue, consistent with an antiscarring effect. However, the collagen fiber network was unchanged. Interestingly, despite the low level of α-smooth muscle actin, TGF-ß3-injected wounds show the same wound volume compared to controls, suggesting that perhaps α-smooth muscle actin expression is more related to granulation tissue remodeling and myofibroblast differentiation than tissue contraction. The absence of TGF-ß3, however, leads to larger and deeper wounds. When stained for α-smooth muscle actin, however, wounds injected with TGF-ß3 neutralizing antibody show very small areas with α-smooth muscle actin positive myofibroblasts at 7 and 11 days post-wounding. Furthermore, collagen fiber network was not fully mature. These results would suggest that TGF-ß3 is required for fibroblast/myofibroblast transdifferentiation and proper granulation tissue maturation in the wound area and are consistent with the effect of the injection of a viral construct containing a mutant TGF-ß3 into cutaneous wounds [26]. Both studies would be consistent with a mathematical model that predicts an increase in wound size after early elimination of TGF-ß [47].

Several reports describe the expression of TGF-ß3 in tissues and cells throughout development and during adulthood, yet not consistently in the same tissues and cells [8], [22], [48], [49]. We took advantage of a new allele with Cre recombinase knocked in the TGF-ß3 locus [27] to determine spatial and temporal expression of Tgfb3 during cutaneous wound healing. X-gal staining indicated the presence of positive signal in the suprabasal layers of the epidermis and hair follicle cells in wounded and unwounded tissues. Although the staining reflects the transformation of cells that have expressed or continue to transcribe from the TGF-ß3 promoter, its pattern in the epidermis and hair follicle is similar to the expression of Cre-recombinase shown previously [31]. These observations only partially mirror previous studies that indicated the presence of Tgfb3 throughout the epidermis, in the granulation tissue and in mesenchymal derivatives [15]. Differences in animal models and method of detection could be at the origin of these discrepancies.

In summary, our study indicates the requirement of an adequate level of TGF-ß3 for proper wound healing. TGF-ß3 is also critical for embryonic development, as *Tgfb3*-deficient mice exhibit cleft palate. It is therefore tempting to postulate that TGF-ß3 is part of a global pathway that is essential for both adult wound repair and embryonic tissue development and that a better understanding of these processes will contribute to the understanding of both wound healing and embryonic development to help us design therapeutic strategies for birth defects and poor healing.
